# Knowledge of primary healthcare workers regarding the prevention and control of non-communicable diseases in Osun State, Nigeria: A rural-urban comparison

**DOI:** 10.4102/phcfm.v13i1.2873

**Published:** 2021-06-29

**Authors:** Adebowale F. Akinwumi, Olapeju A. Esimai, Olusola Fajobi, Ajibola Idowu, Oluwaseun T. Esan, Temitope O. Ojo

**Affiliations:** 1 Department of Community Health, Obafemi Awolowo University Teaching Hospitals Complex, Ile Ife, Nigeria; 2 Department of Community Medicine, Faculty of Clinical Sciences, Ekiti State University, Ado Ekiti, Nigeria; 3 Department of Community Health, Faculty of Clinical Sciences, Obafemi Awolowo University, Ile Ife, Nigeria; 4 Department of Community Medicine, Faculty of Clinical Sciences, Bowen University, Iwo, Nigeria

**Keywords:** non-communicable diseases, primary healthcare, knowledge, healthcare workers, prevention and control

## Abstract

**Background:**

There is a rising burden of non-communicable diseases (NCDs) in the sub-Saharan Africa, and calls for integration of management of selected NCDs with primary healthcare (PHC) have been unrelenting. Cost-effective interventions for the prevention and control of NCDs can be delivered at PHC facilities in low-resource settings by clinical healthcare workers (HCWs).

**Aim:**

This study compared the knowledge of HCWs in PHC facilities regarding the prevention and control of NCDs in rural and urban local government areas (LGAs) of Osun State.

**Setting:**

A comparative cross-sectional study was conducted amongst 400 eligible HCWs recruited using a multistage sampling technique in PHC facilities of six rural and six urban LGAs.

**Methods:**

A pretested self-administered case-scenarios questionnaire was used to assess the knowledge of HCWs regarding the prevention and control of three selected NCDs (diabetes, hypertension and chronic respiratory diseases). Both descriptive and inferential statistics were conducted.

**Results:**

The mean knowledge scores of HCWs regarding the prevention and control of the three NCDs were 17.76 ± 4.41 in rural and 17.62 ± 4.02 in urban LGAs out of 30 maximum scores. The proportion of HCWs with adequate knowledge in the rural LGAs (31.0%) was slightly higher than the urban LGAs (23.0%); however, it was not statistically significant (χ^2^ = 3.247; *p* = 0.072). The major determinants of adequate knowledge include cadre of HCWs, location, years in practice with professional certificate, NCD training course attendance and reported experience managing diabetic patients.

**Conclusion:**

The HCWs in PHC facilities in rural and urban LGAs of Osun State, Nigeria, had a poor knowledge regarding the prevention and control of NCDs. Training and re-training of less-skilled HCWs in the PHC facilities using relevant WHO NCD protocols and guidelines are imperatives to improve their knowledge about the prevention and control of NCDs.

## Introduction

According to the World Health Organization (WHO), the main non-communicable diseases (NCDs) include cardiovascular diseases, diabetes, cancer and chronic respiratory disease.^1,2^ These diseases share four behavioural risk factors: tobacco use, unhealthy diet, physical inactivity and harmful use of alcohol.^2^ The human, social and economic consequences of NCDs are felt by all countries but are particularly devastating in poor and vulnerable populations.^3^

According to a 2018 WHO report, 71% of all global deaths were caused by NCDs, with cardiovascular diseases (31%), cancers (16%) and respiratory diseases, including asthma and chronic obstructive pulmonary disease (7%), being the first, second and third most common causes of global deaths, respectively.^4^ Diabetes constitutes 3% of these global deaths.^4^ Sub-Saharan African countries, including Nigeria, are undergoing epidemiological transition with the decline in the burden of many infectious diseases and a steady rise in the prevalence of NCDs as a major cause of death.^5,6,7,8,9^ According to the 2018 WHO NCDs country profile report, NCDs constituted 29% of the total deaths reported in Nigeria.^4^

Primary healthcare (PHC) has become the bedrock of the country’s health system^10,11^ and constitutes the first point of contact with the health system, especially in rural communities. The care for people with and at risk of NCDs is long term and should be proactive, patient centered, community based and sustainable. Such care can be delivered equitably only through healthcare systems based on PHC.^12,13,14^ Furthermore, the problem of access to quality, low cost and essential NCD interventions is being compounded by the maldistribution of healthcare workers (HCWs) within countries, which varies by locality. The more skilled healthcare workers, especially qualified medical doctors and nurses, tend to concentrate more in the urban areas because of better standards of living and higher salaries compared with the rural regions.^15^

Most of the studies on NCDs in Nigeria had focused on estimating disease burdens and taking inventories of available healthcare facilities.^6,16,17,18,19,20,21,22,23,24^ Only a few had examined the knowledge of HCWs regarding these diseases.^25^ In addition, there is a paucity of comparative studies on the knowledge of HCWs in rural and urban communities of Nigeria about the prevention and control of NCDs. Yet, the sharp distinction in socio-economic characteristics between rural and urban Nigerian communities may be associated with a differential in the knowledge scores of HCWs in the two sub-populations. Also, there may be disparity in their experience and orientation in provision of NCDs’ prevention and care services.

The HCWs required adequate knowledge for early diagnosis and treatment of NCD patients using appropriate technologies at the PHC levels. Thus, a study assessing the knowledge of NCDs amongst HCWs may open a window of opportunities to identify the need for training and re-training of HCWs by providing baseline data on the skill mix of health personnel. This study, therefore, assessed the knowledge of HCWs in PHC facilities in the rural and urban LGAs of Osun State regarding the prevention and control of NCDs and their associated factors.

## Materials and methods

### Study area

The study site is Osun State, one of the 36 states of The Federation of Nigeria, that was created in 1991. It is located in the south-western part of Nigeria, with a projected population of 4.996 million in 2018.^26^ The state is divided into three senatorial districts, namely, Osun Central, Osun East and Osun West. Each of these districts is further divided into two zones. In all, the state has six zones and 30 local government areas (LGAs).^27^ The LGAs are also divided into 16 rural or 14 urban LGAs.^26^ Most of the inhabitants in the state are engaged in farming and trading.^28^

Osun State has 2 tertiary healthcare facilities and 52 secondary healthcare facilities,^28^ with 406 private healthcare facilities in the form of medical centres, hospitals, clinics, and maternity and convalescent homes.^28^ The Executive Director of the State Primary Health Care Development Board (SPHCDB) oversees 762 PHC facilities that are fairly evenly distributed across all the senatorial districts, and rural and urban LGAs in the state. As of October 2020, health personnels from the PHC facilities in the state include 20 doctors, 206 nurses and midwives, 192 Community Health Officers (CHOs), 916 Senior Community Health Extension Workers (CHEWs) and 334 Junior CHEWs.

### Study design

A comparative cross-sectional study design was used for this study. A case scenarios questionnaire was used to obtain information on the level of knowledge of HCWs about diabetes, hypertension and chronic respiratory diseases (three of the four most common NCDs according to the WHO; cancer was excluded because of infrequent presentation of cases at the PHC facilities and limited capacity to manage it).

### Study population

The study population comprised HCWs from the PHC facilities in Osun state, Nigeria. The following cadres of HCWs were included in the study: doctors (where available) and the non-physician HCWs (nurses, CHOs and the Senior CHEWs) who provided consultations for adults at general outpatient units. The exclusion criteria were HCWs who were absent from workplace during the study period and those on leave of absence (≥ 3 months), such as those on terminal leave, maternity leave and study leave.

### Sample size estimation

The minimum sample size for HCWs in this study was calculated using a sample size formula for comparison of two independent proportions.^29^ Considering the result of a similar study conducted in Tanzania, the proportion of HCWs in healthcare centres with satisfactory (adequate) knowledge of the prevention and management of hypertension and diabetes were 65.7% in the urban area and 46.5% in the rural area of northwest Tanzania.^30^ An acceptable margin of error was set at 5%, corrected for an anticipated non-response rate of 10% amongst the respondents, and to allow for a more robust analysis, the minimum sample size of 200 per group was derived giving a total of 400 respondents for the study.

### Sampling techniques

A total of 400 HCWs (200 HCWs in rural PHC facilities and 200 HCWs in urban PHC facilities) were selected using a multistage sampling technique from 33 rural and 33 urban PHC facilities selected from the six rural and six urban LGAs of the state.

In the first stage, two rural and two urban LGAs were selected from each senatorial district (Osun West, Osun Central and Osun East) by simple random sampling (balloting). In the second stage, five or six PHC facilities were selected from each of the selected six rural and six urban LGAs by simple random sampling (balloting). Third stage, the number of PHC workers recruited from each of the selected healthcare facilities in rural and urban LGAs was proportionate to the size of eligible HCWs in the facilities. The eligible HCWs were stratified by job cadre. The number of doctors, nurses, CHOs and senior CHEWs selected from each facility was determined proportionate to the total number of HCWs in each cadre from the facility. The eligible HCWs in each stratum (job cadre) were selected using a systematic sampling technique. A list of HCWs in each of the strata (job cadres) was obtained; the number of healthcare workers in the stratum was divided by the desired number to obtain the sampling interval. The first respondent was selected by simple random sampling (through balloting from one to the sampling interval number). On average, every second eligible HCW in each stratum was selected, except for medical doctors where most LGAs did not have more than one, and total sampling method was employed.

### Data collection tools

This was a self-administered questionnaire adapted from a case-scenario questionnaire used by Peck et al.^30^ to assess the preparedness (knowledge and experience) of HCWs in order to manage hypertension and diabetes under PHC in Tanzania. This instrument has been standardised and used in another study.^31^ The case-scenario questions for chronic respiratory diseases were developed using the template by Peck et al.^30^ This instrument collected information on the background characteristics of respondents, knowledge of HCWs regarding the prevention and control of selected NCDs, provision of NCD-related services and attendance at any in-service courses or training on common NCD management.

### Pre-testing of research instrument

The questionnaire was pretested in six PHC facilities from Ife East LGA, which were not selected for the main study, using at least 10% of sample size calculated. Eligible HCWs were selected using a systematic sampling technique amongst the doctors, nurses, CHOs and senior CHEWs. This helped us to address areas of ambiguity in the questionnaires and to determine appropriateness of each question in eliciting the required responses.

### Data collection procedure

Six research assistants were involved in the data collection. They participated in a 2-day training course on the content and administration of the research instrument. Data were collected between 26 February 2018 and 13 April 2018 by the research assistants under supervision of the lead researcher.

### Measurement and scoring

The key outcome variable of this study, the knowledge of HCWs about the prevention and control of NCDs, was scored appropriately. The knowledge of healthcare workers regarding the prevention and management of selected NCDs - diabetes, hypertension and chronic respiratory diseases (e.g. asthma) - was assessed using case scenarios containing 10-item questions per each NCD considered.

A score of 1 was assigned to each correct response, with a total score of 10 per case scenario. The composite score of knowledge of the HCWs on each NCD was categorised into good knowledge (9–10), fair knowledge (7–8) and poor knowledge (≤ 6).^30^ The composite score of knowledge of the HCWs on the NCDs using the three case scenarios with a total score of 30 was categorised into good knowledge (26–30), fair knowledge (21–25) and poor knowledge (≤ 20).

The knowledge scores of the HCWs were further dichotomised into adequate (being knowledgeable) and inadequate knowledge (not knowledgeable). Adequate knowledge (7–10/10; 21–30/30) referred to good or fair knowledge and inadequate knowledge (≤ 6/10; ≤ 20/30) referred to poor knowledge. This dichotomous categorisation is because the score of ≥ 7 per case scenario of NCD and ≥ 21 for the three selected NCDs were adjudged reasonable for the HCWs to offer effective essential NCD care.^30,31^

### Data analysis

Data obtained were entered into a spread sheet using EpiData software, version 3.1, and analysed using STATA statistical software, version 14. Categorical variables were summarised using proportions and presented in appropriate tables and charts. Continuous variables were summarised using mean and standard deviation (s.d.). At the bivariate level, independent samples *t*-test, *F*-test and chi-square test (or Fisher’s exact test where applicable) were carried out to determine the relationship between two variables as appropriate. Binary logistic regression analysis was carried out to identify the major determinants of knowledge of HCWs in PHC facilities regarding the prevention and control of NCDs. The level of significance was determined at *p* < 0.05. Adjusted odds ratio (OR) and 95% confidence interval (CI) were obtained to identify the determinants of knowledge of prevention and control of NCDs amongst the study population.

### Ethical considerations

Ethical approval was obtained from the Health Research and Ethics Committee, Ministry of Health, Osun state, Nigeria (OSHREC/PRS/569T/127) prior to commencement of the study. A written informed consent was obtained from each participant after adequate information was provided on the objectives of the study, the risk and benefits. Participation of respondents was voluntary; confidentiality and data security were assured.

## Results

Two hundred eligible HCWs from PHC facilities in six rural LGAs and 200 from six urban LGAs in Osun State participated in this research study. The response rate was estimated to be 100% because questionnaires were filled and returned immediately to the research assistants, who, in turn, scrutinised these questionnaires for completeness, and the lead researcher cross-checked the questionnaires before leaving the field on each day of data collection.

Of the HCWs, 90.0% in the rural and 89.0% in the urban LGAs have been working for 10 years or more in the PHC system. The socio-demographic characteristics of the HCWs from PHC facilities in the rural and urban LGAs were comparable; however, there was no statistically significant difference across the age group, sex and duration of years in PHC services (Table 1).

**TABLE 1:** Distribution of Respondents by their Socio-Demographic Characteristics

Socio-demographic characteristics of health care workers	Location						Statistics			
	Rural (*n* = 200)			Urban (*n* = 200)			LR	*df*	*p*	χ2
	*n*	%	Mean	*n*	%	Mean				
**Age (years)**	-	-	44.1 ± 6.5	-	-	43.7 ± 6.6	0.472	3	0.925	-
20-29	3	1.5	-	3	1.5	-	-	-	-	-
30-39	51	25.5	-	50	25.0	-	-	-	-	-
40-49	100	50.0	-	106	53.0	-	-	-	-	-
50 and above	46	23.0	-	41	20.5	-	-	-	-	-
Range (years)	25–60	-	-	20–58	-	-	-	-	-	-
**Sex**	-	-	-	-	-	-	-	1	0.727	0.122
Male	19	9.5	-	17	8.5	-	-	-	-	-
Female	181	90.5	-	183	91.5	-	-	-	-	-
**Cadre of health workers**	-	-	-	-	-	-	-	3	0.196	4.687
Medical doctor	6	3.0	-	4	2.0	-	-	-	-	-
Registered nurse/public health nurse	23	11.5	-	31	15.5	-	-	-	-	-
CHO	33	16.5	-	45	22.5	-	-	-	-	-
Senior CHEW	138	69.0	-	120	60.0	-	-	-	-	-
**Duration of completed years in practice with professional certificate**	-	-	16.2 ± 7.5		15.6±7.6	-	-	2	0.474	1.493
0–9	30	15.0	-	39	19.5	-	-	-	-	-
10–19	107	53.5	-	99	49.5	-	-	-	-	-
20 and above	63	31.5	-	62	31.0	-	-	-	-	-
**Duration of completed years working in the PHC system**	-	-	16.4 ± 6.4	-	-	16.3 ± 6.6	-	2	0.678	0.778
0–9	21	10.5	-	22	11.0	-	-	-	-	-
10–19	117	58.5	-	124	62.0	-	-	-	-	-
20 and above	62	31.0	-	54	27.0	-	-	-	-	-

Note: The mean ages of respondents were 44.09 ± 6.54 years in the rural and 43.73 ± 6.64 years in the urban LGAs.

χ2, chi-square test; *df*, degree of freedom; LR, likelihood ratio; CHO, Community Health Officer; CHEW, Community Health Extension Worker; PHC, primary health care.

A comparison of mean knowledge scores of HCWs on the prevention and control of selected NCDs from PHC facilities in the rural and urban LGAs using an independent sample *t*-test was made (Table 2). The mean knowledge scores on the prevention and control of hypertension (6.24) amongst HCWs from PHC facilities in rural LGAs were slightly higher than those (6.19) amongst HCWs from the PHC facilities in urban LGAs. Amongst the three selected NCDs, the mean knowledge scores of prevention and control of the NCDs amongst the HCWs were lowest for diabetes mellitus, 5.5 ± 1.8 in the rural LGAs and 5.4 ± 1.7 in the urban LGAs. The mean knowledge scores (17.8/17.6) of HCWs on prevention and control of the three NCDs in PHC facilities in the rural and urban LGAs were similar; however, no statistically significant difference was observed (*t* = 0.332; *p* = 0.740).

**TABLE 2:** Comparison of mean knowledge scores of healthcare workers on the prevention and control of selected non-communicable diseases from primary healthcare facilities in rural and urban local government areas.

Knowledge scores of HCWs on prevention and control of NCDs in PHC facilities	*n*	Mean	s.d.	95% CI	Statistic *t*-test	*p*
**Hypertension**						
Rural	200	6.2	1.9	6.0–6.5	0.299	0.765
Urban	200	6.2	1.8	5.9–6.4	-	-
**Diabetes mellitus**						
Rural	200	5.5	1.8	5.3–5.8	0.870	0.385
Urban	200	5.4	1.7	5.1–5.6	-	-
**Chronic respiratory diseases**						
Rural	200	6.0	1.6	5.8–6.2	−0.435	0.664
Urban	200	6.1	1.6	5.9–6.3	-	-
**Overall**						
Rural	200	17.8	4.4	17.1–18.4	0.332	0.740
Urban	200	17.6	4.0	17.1–18.2	-	-

s.d., standard deviation; CI, confidence interval; HCWs, healthcare workers; NCDs, non-communicable diseases; PHC, primary healthcare; LGA, local government areas.

The comparison of knowledge of healthcare providers regarding the prevention and control of NCDs in the rural and urban PHC facilities is shown (Table 3). The HCWs in these facilities demonstrated an inadequate (poor) knowledge of the prevention and control of the three NCDs: 150 (75.0%) for diabetes amongst HCWs in urban LGAs and 142 (55.0%) for hypertension amongst the rural healthcare providers. Overall, the proportion of HCWs with inadequate knowledge of the prevention and control of NCDs in PHC facilities was higher in the urban LGAs 154 (77.0%) than in the rural LGAs 138 (69.0%); however, there was no statistically significant difference (χ^2^ = 3.247; *p* = 0.072).

**TABLE 3:** Comparison of knowledge of healthcare workers on the prevention and control of non-communicable diseases in rural and urban primary health care facilities.

Categories of knowledge scores of HCWs on selected NCDs	Location				Statistics	
	Rural (*n* = 200)		Urban (*n* = 200)		*χ*2	*p*
	*n*	%	*n*	%		
**Hypertension**						
Adequate	90	45.0	83	41.5	0.480	0.499
Inadequate	110	55.0	117	58.5	-	-
**Diabetes mellitus**						
Adequate	58	29.0	50	25.0	0.812	0.368
Inadequate	142	71.0	150	75.0	-	-
**Chronic respiratory diseases**						
Adequate	85	42.5	89	44.5	0.163	0.687
Inadequate	115	57.5	111	55.5	-	-
**Overall**						
Adequate	62	31.0	46	23.0	3.247	0.072
Inadequate	138	69.0	154	77.0	-	-

HCWs, healthcare workers; NCDs, non-communicable diseases.

The categorisation of the knowledge scores of different cadres of HCWs on prevention and control of the three selected NCDs in PHC facilities is discussed (see Table 4). In both rural and urban LGAs, all the 10 medical doctors (100.0%) have adequate knowledge regarding the prevention and control of the three NCDs. Amongst the non-physician HCWs, the nurses had the best knowledge about the prevention and control of NCDs. Of the nurses, 16 (70.0%) from rural LGAs and 17 (55.0%) from urban LGAs had adequate knowledge of the prevention and control of the three NCDs. The senior CHEWs had the least proportion of its cadre with adequate knowledge of the prevention and control of the three NCDs; only 25 (18.0%) in the rural LGAs and 13 (11.0%) in the urban LGAs. The differences across the cadres in both the rural and urban LGA settings were statistically significant (*p* ≤ 0.001; *p* ≤ 0.001).

**TABLE 4:** Categorisation and comparison of knowledge of different cadres of healthcare workers on prevention and control of the three non-communicable diseases in rural and urban primary healthcare facilities.

Knowledge of the three NCDs	Doctors		Nurses		CHO		Senior CHEW		Likelihood ratio	*p*
	*n*	%	*n*	%	*n*	%	*n*	%		
**Rural**										
Adequate	6	100.0	16	69.6	15	45.5	25	18.1	**43.310**	< 0.001*
Inadequate	0	0.0	7	30.4	18	54.5	113	81.9	-	-
**Urban**										
Adequate	4	100.0	17	54.8	12	26.7	13	10.8	38.510	< 0.001*
Inadequate	0	0.0	14	45.2	33	73.3	107	89.2	-	-

NCDs, non-communicable diseases; CHO, Community Health Officer; CHEW, Community Health Extension Worker.

* , Statistically significant at *p* < 0.05.

A comparison of the mean composite knowledge scores of different cadres of HCWs in PHC facilities on the three diseases using analysis of variance (ANOVA) is made (Table 5). Medical doctors have the highest mean composite knowledge score (rural LGAs = 27.5; urban LGAs = 26.8), followed by the nurses (rural LGAs = 21.5; urban LGAs = 20.9), whilst the senior CHEWs in PHC facilities of the urban LGAs have the least (rural LGAs = 16.4; urban LGAs = 16.3). The comparison of mean composite knowledge scores of HCWs on three NCDs across the four cadres (medical doctors, registered nurses, CHOs and Senior CHEW) of HCWs was significantly different across the groups (rural LGAs: *F* = 28.41, *p* < 0.001; urban LGAs: *F* = 25.73, *p* < 0.001).

**TABLE 5:** Comparison of mean composite knowledge scores of healthcare workers on prevention and control of the three non-communicable diseases across various cadres of healthcare workers.

Cadres of HCWs	*n*	Mean	s.d.	*df*	*F*†	*p*
**Rural**						
Medical doctors	6	27.5	1.1	3; 196	28.41	< 0.001*
Nurse or public health nurses	23	21.5	3.2	-	-	-
CHO	33	18.9	4.0	-	-	-
Senior CHEW	138	16.4	3.8	-	-	-
Total	200	17.8	4.4	-	-	-
**Urban**						
Medical doctors	4	26.8	2.1	3; 196	25.73	<0.001*
Nurse or public health nurses	31	20.9	3.0	-	-	-
CHO	45	18.2	3.6	-	-	-
Senior CHEW	120	16.3	3.5	-	-	-
Total	200	17.6	4.0	-	-	-

HCWs, healthcare workers; CHO, Community Health Officer; CHEW, Community Health Extension Worker; s.d., standard deviation; df, degree of freedom.

* , Statistically significant at *p* < 0.05.

† , Analysis of variance (ANOVA) used.

The binary logistic regression of factors associated with adequate knowledge of HCWs regarding the prevention and control of the three selected NCDs in PHC facilities is listed (see Table 6). The determinants of the level of knowledge include the cadre of HCWs (doctors were excluded because they all had adequate knowledge) (nurses OR: 11.6, 95% CI: 5.48–24.54; CHOs OR: 2.96, 95% CI: 1.52–5.76), location (urban OR: 0.46, 95% CI: 0.27–0.80), years in practice with professional certificate (10–19 years OR: 0.41, 95% CI: 0.19–0.87), NCD training course attendance (never attended any NCD course OR: 0.42, 95% CI: 0.18–0.98), reported availability of drugs for hypertension in the PHC facility (availability of some drugs for management of hypertension OR: 5.52, 95% CI: 1.65–18.40) and reported number of diabetic patients seen in the last 3 months (1–5 patients, OR: 2.44, 95% CI: 1.33–4.51).

**TABLE 6:** Binary logistic regression of factors associated with adequate knowledge of healthcare workers regarding prevention and control of the three selected non-communicable diseases in primary healthcare facilities.

Variables	Odd ratio	95% CI	*p*
**Sex**			
Male (Ref)	1.0	-	-
Female	0.70	0.26–1.90	0.484
**Cadre of HCWs**			
Senior CHEWs (Ref)	1.0	-	-
CHOs	2.96	1.52–5.76	0.001*
Nurses/PH nurses	11.60	5.48–24.54	< 0.000*
**Location**			
Rural (Ref)	1.0	-	-
Urban	0.46	0.27–0.80	0.006*
**Duration of years in practice with certificate**			
0–9 years. (Ref)	1.0	-	-
10–19 years	0.41	0.19–0.87	0.020*
20 >	0.79	0.36–1.75	0.573
**NCD training course undergone by HCWs**			
< 1 year ago (Ref)	1.0	-	-
> 1 year ago	0.66	0.29–1.51	0.322
Never	0.42	0.18–0.98	0.044*
**Senatorial districts**			
Osun West (Ref)	1.0	-	-
Osun Central	1.57	0.82–3.01	0.173
Osun East	1.02	0.52–2.01	0.944
**Reported availability of drugs for hypertension**			
All the drugs are available (Ref)	1.0	-	-
Some of the drugs are available	5.52	1.65–18.40	0.005*
No drug is available	1.97	0.69–5.59	0.205
**Reported number of diabetic patients seen in the last 3 months**			
None (Ref)	1.0	-	-
1–5 patients	2.44	1.33–4.51	0.004*
> 5 patients	1.12	0.21–5.98	0.894

HCWs, healthcare workers; CHO, Community Health Officer; CHEW, Health Extension Worker; NCDs, non-communicable diseases; CI, confidence interval; PH, public health; Ref, reference.

* , Statistically significant.

## Discussion

This research study showed that HCWs had a poor knowledge of the prevention and control of NCDs in rural and urban PHC facilities in Nigeria. Several studies in many sub-Saharan African countries, such as the Democartic Republic of Congo, Uganda, Tanzania, South Africa and Nigeria, have also reported similar findings of poor knowledge of prevention and care of selected NCDs, especially diabetes and hypertension, amongst HCWs in low-level healthcare settings.^30,31,32,33,34,35^ Similar proportions (about half) of HCWs had adequate knowledge (good or fair knowledge) of prevention and control of hypertension and chronic respiratory diseases, and only a quarter were knowledgeable about diabetes mellitus in both rural and urban LGAs. This was in contrast with the findings of a study conducted in Tanzania, which suggested that HCWs in the urban areas had a better knowledge of the prevention and management of hypertension and diabetes mellitus than those in the rural areas.^30^ The reasons for the overall poor knowledge of HCWs in this study might be because of inadequate training of HCWs on the prevention and control of NCDs, and poor availability of guidelines and IEC materials for selected NCDs. These inadequacies have also been reported in another study in Nigeria where HCWs in PHC facilities had limited participation in NCD capacity-building activities and outreach programmes.^36^

The finding of a slightly higher proportion of HCWs with adequate knowledge regarding the prevention and control of the three NCDs in the rural LGAs than in the urban LGAs, although not significant, might be because of the deployment and transfer of HCWs across rural and urban LGAs settings without restriction. Also, PHC facilities are the major providers of healthcare services in the rural LGAs, and the HCWs in these facilities may likely need to offer essential interventions for selected NCDs because of the limited options for referral to higher levels within their communities. This is unlike in the urban LGAs where higher levels of care, such as general hospitals, tertiary or specialist hospitals, and better equipped private healthcare facilities, likely abound, and patients may report first at those facilities to seek care for selected NCDs than at the PHC centres. This may influence the knowledge and experience of HCWs regarding the prevention and management of NCDs in the PHC facilities in both LGA settings.

The medical doctors in the PHC facilities had better knowledge of prevention and control of selected NCDs than other cadres of HCWs. All the doctors in PHC facilities in both rural and urban LGAs also were knowledgeable about the prevention and control of the three selected NCDs. This was similar to the report of studies by Peck et al.^30^ in Tanzania and by Katende et al.^31^ in Uganda, which suggest that medical doctors had better knowledge of NCDs than non-physician clinicians, nurses and assistants. According to several studies in low-income and middle-income countries, despite their good knowledge among doctors, they had a minimal impact on the control of NCDs in the PHC facilities because of their lower numbers compared to other HCWs at the PHC facilities.^36,37^ The situation in our study area was also similar.

The current study showed that nurses have better knowledge of prevention and control of NCDs amongst the non-physician healthcare workers (NPHWs) in the PHC facilities than CHOs and senior CHEWs. This finding was corroborated by the report of a study, which revealed that nurses had a better knowledge of screening for diabetes amongst NPHWs in PHC facilities across Nigeria.^38^ The finding in the current study might have a positive implication on the task shifting policy and drive of the government to allow nurses perform specific roles in the prevention and care of selected NCDs at the PHC level.^39^

The findings of inadequate (poor) knowledge of HCWs in PHC facilities in rural and urban LGAs on the prevention and control of NCDs have implications on the delivery of effective essential NCD interventions to the people within various communities where the facilities are located. These might include inadequate health promotion and prevention activities for NCDs, inaccurate diagnosis, poor management of non-complicated and non-referral of complicated selected NCDs conditions amongst the people. In the long term, this might lead to the late presentation and early onset of complications amongst people with NCDs, with possible attendant catastrophic health expenditures.

The findings of the present study revealed that cadre of HCWs, location, years in practice with professional certificate, NCD training course attendance and the reported number of diabetic patients seen in the last 3 months were the major determinants of adequate knowledge of prevention and control of NCDs amongst HCWs in the PHC facilities. Alotaibi et al.^40^ in their integrative review of studies on diabetes knowledge amongst nurses reported similar findings of nurses’ inadequate training and education about diabetes, lack of access to relevant knowledge-based resources and work experience being major factors that influenced diabetes knowledge of nurses in developed and developing countries. Also, in the Democratic Republic of Congo, the level of knowledge of therapeutic objectives of treatment and control of diabetes was related to the level of healthcare providers’ training and level of responsibilities at their health facilities.^32^

### Study limitations

The self-reported nature of the responses might introduce self-report and social desirability biases. This was minimised by assuring the respondents of absolute confidentiality. This study is not generalisable to the whole country and other HCWs apart from PHC workers.

## Conclusion

The HCWs in PHC facilities in the rural and urban LGAs of Osun State have poor knowledge about the prevention and control of selected NCDs. The major determinants of adequate knowledge include the cadre of HCWs, location, years in practice with professional certificate, NCD training course attendance and reported number of diabetic patients seen in the last 3 months. The authors thus recommend that the SPHCDB should embark on training and re-training of NPHWs in PHC facilities in the rural and urban LGAs on prevention and control of the selected NCDs using relevant WHO protocols and guidelines. It is also imperative to recruit more skilled HCWs, especially medical doctors and nurses, who will be responsible for conducting regular supportive supervisions for the less-skilled healthcare personnel. This will equally improve the human resources for health mix in the PHC facilities of Osun state, Nigeria.
